# Polymorphism in the HASPB Repeat Region of East African *Leishmania donovani* Strains

**DOI:** 10.1371/journal.pntd.0002031

**Published:** 2013-01-24

**Authors:** Arie Zackay, Abdelmajeed Nasereddin, Yegnasew Takele, Dagimawie Tadesse, Workagegnehu Hailu, Zewdu Hurissa, Sisay Yifru, Teklu Weldegebreal, Ermias Diro, Aysheshm Kassahun, Asrat Hailu, Charles L. Jaffe

**Affiliations:** 1 Department of Microbiology and Molecular Genetics, IMRIC, Hebrew University-Hadassah Medical School, Jerusalem, Israel; 2 Leishmaniasis Research and Treatment Centre, University of Gondar, Gondar, Ethiopia; 3 Leishmaniasis Research and Treatment Centre, Arba Minch Hospital, Arba Minch, Ethiopia; 4 Department of Parasitology, Charles University in Prague, Prague, Czech Republic; 5 School of Medicine, College of Health Sciences, Department of Microbiology, Immunology and Parasitology, Addis Ababa University, Addis Ababa, Ethiopia; Lancaster University, United Kingdom

## Abstract

**Background/Objectives:**

Visceral leishmaniasis (VL) caused by *Leishmania donovani* is a major health problem in Ethiopia. Parasites in disparate regions are transmitted by different vectors, and cluster in distinctive genotypes. Recently isolated strains from VL and HIV-VL co-infected patients in north and south Ethiopia were characterized as part of a longitudinal study on VL transmission.

**Methodology/Principal Findings:**

Sixty-three *L. donovani* strains were examined by polymerase chain reaction (PCR) targeting three regions: internal transcribed spacer 1 (ITS1), cysteine protease B (cpb), and HASPB (k26). ITS1- and cpb - PCR identified these strains as *L. donovani*. Interestingly, the k26 - PCR amplicon size varied depending on the patient's geographic origin. Most strains from northwestern Ethiopia (36/40) produced a 290 bp product with a minority (4/40) giving a 410 bp amplicon. All of the latter strains were isolated from patients with HIV-VL co-infections, while the former group contained both VL and HIV-VL co-infected patients. Almost all the strains (20/23) from southwestern Ethiopia produced a 450 bp amplicon with smaller products (290 or 360 bp) only observed for three strains. Sudanese strains produced amplicons identical (290 bp) to those found in northwestern Ethiopia; while Kenyan strains gave larger PCR products (500 and 650 bp). High-resolution melt (HRM) analysis distinguished the different PCR products. Sequence analysis showed that the k26 repeat region in *L. donovani* is comprised of polymorphic 13 and 14 amino acid motifs. The 13 amino acid peptide motifs, prevalent in *L. donovani*, are rare in *L. infantum*. The number and order of the repeats in *L. donovani* varies between geographic regions.

**Conclusions/Significance:**

HASPB repeat region (k26) shows considerable polymorphism among *L. donovani* strains from different regions in East Africa. This should be taken into account when designing diagnostic assays and vaccines based on this antigen.

## Author Summary

HASPB belongs to a hydrophilic repeat-containing surface antigen family found in *Leishmania*. The *L. infantum*/*L. donovani* protein has been used for diagnosis of visceral leishmaniasis, and is a putative vaccine candidate for this disease. Visceral leishmaniasis is a fatal disease, and approximately one third of the cases are found in East Africa. The k26 – PCR, which amplifies the repeat region of HASPB, produced different amplicon sizes for recent Ethiopian *L. donovani* depending on the strain's geographic origin. Further analysis showed that the number and order of the peptide motifs, either 13 or 14 amino acids long, comprising the *L. donovani* repeats varies between endemic regions of East Africa. Polymorphism in the amino acid sequence of the peptides was also observed. In addition, the 13 amino acid peptide motifs prevalent in *L. donovani* are rare in *L. infantum*. The observed polymorphisms in the HASPB repeat region suggests that custom antigens may be needed for diagnosis or vaccination in distinct endemic foci.

## Introduction

Parasites belonging to the *Leishmania donovani* complex, *L. donovani* and *L. infantum* (synonym = *L. chagasi*), are the main causative agents of visceral leishmaniasis (VL), also known as kala-azar. This disease is invariably fatal if not properly diagnosed and treated. The World Health Organization (WHO) estimates that the yearly incidence of VL is between 2–400,000 cases, resulting in 20–40,000 deaths annually with the majority of cases, >90%, occurring in Brazil, the Indian subcontinent and east Africa [1]. VL in the latter region is found primarily in Sudan, South Sudan and Ethiopia where an estimated 30,000–57,000 cases occur each year [1], [2], [3]. In East Africa and India, VL is primarily caused by *L. donovani*, and believed to be an anthroponosis, while in other regions, where VL is caused by *L. infantum*, this disease is a zoonosis with dogs and wild canids acting as reservoir hosts [4].

In Ethiopia, VL is distributed throughout the lowlands with the most important foci found in northwestern and southwestern parts of the country. However, the ecology, vectors responsible for parasite transmission, and epidemiology of VL differ between these regions. Northwestern Ethiopia (NW) accounts for ∼60% of the VL cases [3], and a majority of the HIV - VL co-infections, with the disease focused in the Metema - Humera region near the Sudanese border. This is a semi-arid region, with extensive commercial monoculture, and scattered Acacia - Balanite forests. *Phlebotomus orientalis* is the suspected vector responsible for transmission [3], [5]. The recent large increase in VL in NW Ethiopia has been correlated with agricultural development, and the large influx of seasonal workers ([6], [7]. Migrant workers returning from this area to the non-endemic highlands appear to be responsible for introducing the VL into the latter regions, as typified by the recent outbreak that occurred in Libo-Kemkem, South of Gondar [6]. In southwestern Ethiopia (SW), VL foci are mainly located in the Omo River plains, Segen and Woito Valleys, and near the border with Kenya [3], [8]. These regions include savannah and forest, and *P. martini* and *P. celiae* have been implicated as vectors [3], [9], [10]. Disease in Southern Ethiopia appears to be sporadic and stable occurring most frequently among children or young adults [8].

Analysis of parasites belonging to the *L. donovani* complex using multiple molecular markers that included DNA sequences of protein coding, non-coding and intergenic regions, microsatellites (MLMT) and other techniques, resulted in a revised taxonomy [11]. East African strains, previously split into *L. donovani*, *L. archibaldi* or *L. infantum* by multilocus enzyme electrophoresis (MLEE) are now classified in one group as *L. donovani s.s*. This large study confirmed several earlier publications using individual molecular techniques [12], [13], [14]. Several of these studies identify genetically distinct populations among the *L. donovani* complex associated with different geographic regions [13], [14]. Recently, analysis using 14 unlinked microsatellite markers of 90 East African strains, including 63 new isolates from Ethiopia, showed that *L. donovani* can be divided into two genetically distinct populations, Sudan plus NW, and Kenya plus SW. These major groups could also be further divided into several subpopulations [15]. Although MLMT easily distinguishes between two main *L. donovani* genotypes in Ethiopia, NW and SW type, and can produce individual parasite genetic pedigrees, it is relatively expensive, requires more sophisticated analysis, and not available in most laboratories working on *Leishmania*.

*HASPB* (hydrophilic acylated surface protein B) belongs to a family of orthologous genes, originally called the LmcDNA16 locus, found in Old and New World *Leishmania* species [16]. The protein is expressed only by metacyclic promastigotes and amastigotes, and is characterized by amino acid repetitive domains that show both inter- and intra-species polymorphism [17], [18], [19]. A recent study using *L. major* LmcDNA16 locus null mutants, and parasites complemented for either HASPB or the whole locus showed that this protein is involved in metacyclogenesis and promastigote localization in the sand fly vector [20]. The repeat region of the *L. donovani* and *L. infantum* HASPB protein, also known as k26, is recognized by human and canine VL sera, and has been used with varying success for serodiagnosis [19], [21], [22], [23], [24], [25], [26]. In addition, HASPB has been shown to be a potential vaccine candidate [27], [28], [29].

A specific PCR targeting the *L. donovani* complex HASPB repeat region (k26 – PCR) was shown to distinguish between *L. donovani* and *L. infantum* strains grouping them according to the size of the amplicon [30]. However, only a few East African strains from Sudan (n = 6) and Ethiopia (n = 2) isolated between 1954 and 2000 were examined. More recently, Gadisa et al. [31] characterize five clinical isolates from VL patients in Ethiopia by k26 - PCR. Only a single PCR fragment was observed, all the same size as the WHO reference strain LV9 (MHOM/ET/67/HU3).

In this study, we characterized 63 recent *L. donovani* strains from Ethiopia using k26 - PCR, and high resolution melt (HRM) analysis. Several strains from Kenya, Sudan and India were also included for comparison. Analysis by these techniques split the Ethiopian strains into groups that are correlated with the geographic origin of the parasite strain. DNA sequencing of the amplicons showed that the number and organization of the peptide motifs comprising the *L. donovani* HASPB repeat domain varies with the geographic origin of the strain. Potential effect of k26 polymorphism on use of HASPB for serodiagnosis and vaccination is discussed.

## Materials and Methods

### Ethical considerations

This study was conducted according to the Helsinki declaration, and was reviewed and approved by the Institutional Review Board (IRB), Medical Faculty, Addis Ababa University. Written informed consent was obtained from each study participant.

### Clinical isolates and reference strains used in the study

**Figure 1:**
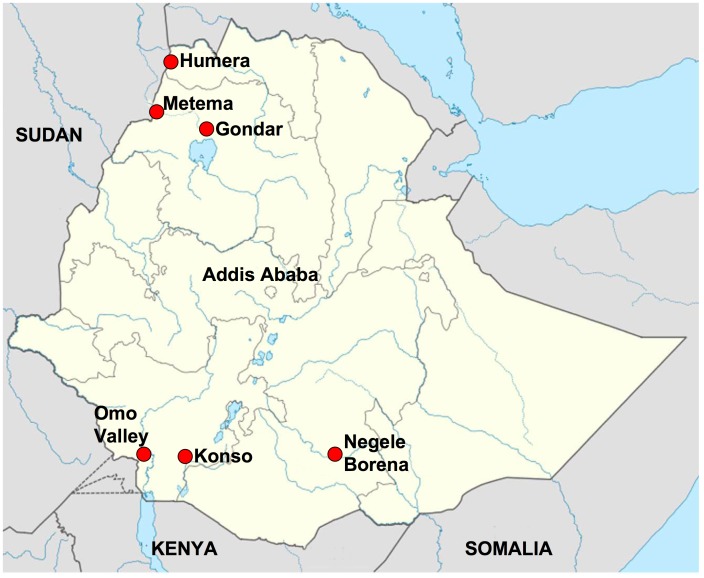
Origin of Ethiopian *Leishmania donovani* strains used in this study. Specific details on all strains are provided in Table S1.

*Leishmania* strains (n = 63) recently isolated from patients with VL or HIV - VL co-infections in northwestern (n = 40) and southern Ethiopia (n = 23), see Figure 1, were cultured in M199/Hepes pH 6.8 medium supplemented with 10% fetal calf serum and antibiotics [32]. DNA extraction was carried out using the Gentra DNA extraction kit (Gentra system, Minneapolis, MN). In addition, DNA from *L. donovani* strains, Ethiopian (n = 24) and Kenyan (n = 7) previously examined by MLMT [15], and from Sudan (n = 2) and India (n = 2) was also analyzed. The strains used in this study are described in Table S1.

### Polymerase chain reaction (PCR) and high resolution melt (HRM) analysis

Internal transcribed spacer 1 (ITS1) - PCR followed by restriction fragment length polymorphism (RFLP) analysis was carried out as described [33]. A modified, “short” cpbE/F - PCR was used to distinguish between *L. infantum* and *L. donovani*, and was carried out using the primers 5-GTTATGGCTGCGTGGCTTG-3 (this study) and 5-CGTGCACTCGGCCGTCTT-3 [34]. DNA (50–100 ng) was added to a PCR - Ready Supreme reaction mix (Syntezza Bioscience, Jerusalem, Israel) in 25 µL total reaction, and performed as follows: Initial denaturation 4 min at 95°C; followed by 35 cycles with each cycle consisting of denaturation 30 s at 94°C, annealing 15 s at 50°C, and extension 60 s at 72°C. Final extension step was carried out for 10 min at 72°C. PCR products were separated by 2% agarose gel electrophoresis, stained with ethidium bromide and visualized using UV light. *L. infantum* gives a 361 bp product, while *L. donovani* give a 400 bp product in the short cpbE/F PCR.

K26 - PCR was carried out as described [30], and analyzed by agarose gel electrophoresis as above. HRM analysis of the k26 amplicons was carried out as follows: DNA (20 ng) or no DNA control was added to Type-it HRM PCR Kit reaction mix (12.5 µl, QIAGEN GmbH, Germany) containing the k26 primers (1 µM each final concentration), and ultra-pure PCR-grade water (final volume 25 µl/PCR). Amplification conditions were as follows: 10 min denaturation at 95°C, followed by 40 cycles of denaturation 5 s at 95°C; annealing 10 s at 55°C; and extension 20 s at 72°C. HRM ramping was carried out at 0.2°C/s from 70 to 95°C. HRM PCR and analysis were performed using a Rotor-Gene 6000 real-time thermal analyzer (Corbett Life Science, Australia). Positive-control (reference strain DNA, 20 ng/reaction) and negative-control reactions were included in each experiment. A normalized melt window, ∼85 to 90°C, was used in analyzing the HRM curves.

### DNA sequencing and analysis

For direct sequencing, the PCR products were purified using Wizard SV gel and PCR clean-up system purification kit (Promega, WI, USA). The eluted DNA was sequenced at the Center for Genomic Technologies, The Hebrew University of Jerusalem, and the sequences submitted to GeneBank at NCBI. Peptide sequences were obtained using the ExPASy Translate Tool (http://web.expasy.org/translate/). DNA and peptide sequences were aligned using CLUSTAL 2.1 (http://www.ebi.ac.uk/Tools/msa/clustalw2), and linear B-cell epitopes predicted using BepiPred and ABCpred (http://www.cbs.dtu.dk/services/BepiPred and http://www.imtech.res.in/raghava/abcpred/index.html, respectively) [35] [36].

## Results and Discussion

### Characterization of recent *Leishmania* isolates from Ethiopian patients with visceral leishmaniasis

**Figure 2:**
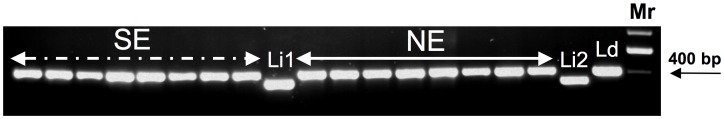
Characterization of Ethiopian parasite strains from patients with visceral leishmaniasis by short cpbE/F - PCR. Amplicons were separated by electrophoresis on 2% agarose gel and staining with ethidium bromide. Reference DNA samples for *Leishmania infantum* are indicated by Li1 (MCAN/IL/2000/LRC-L792) and Li2 - (MHOM/TN/1980/IPT1), and for *L. donovani* by Ld (MHOM/SD/1962/1S cl2). Mr −100 bp molecular weight marker. Representative parasite DNA samples examined by short cpbE/F - PCR from left to right Southern Ethiopia (SE): AM546, AM548, AM551, AM552, AM553, AM554, AM560, AM563 and Northern Ethiopia (NE): GR284, GR353, GR356, GR358, GR361, GR378, GR379, GR383.

DNA was purified from 63 *Leishmania* strains isolated from Ethiopian patients presenting with either VL or HIV-VL co-infections. As an initial step the DNA's were first examined by ITS1 - PCR RFLP, and shown to belong to the *L. donovani* complex (data not shown). Since it can be difficult to distinguish between *L. infantum* and *L. donovani* using the ITS1 - PCR RFLP [33], we also analyzed these strains using a modified cpbE/F – PCR based on the procedure described by Hide and Banuls [34]. The *L. infantum cpbE* and *L. donovani cpbF* genes are similar except for a 39 bp insert only present in the latter species. This difference is more easily observed by gel electrophoresis using the short cpbE/F – PCR where the amplicon size is 361 bp for *L. infantum* and 400 bp for *L. donovani*, rather than 702 and 741 bp, respectively, in the original procedure [34], since the relative size difference between the two short PCR products is larger. This alleviates the need for additional treatments, such as digestion with restriction enzymes [31], [37], which can facilitate species identification. Using the short cpbE/F – PCR all 39 new Ethiopian VL patient strains gave 400 bp PCR products typical of *L. donovani* (Figure 2, and data not shown), and are identical to the Sudanese reference strain (MHOM/SD/1962/1S cl2, lane Ld). As expected, the *L. infantum* reference strains (MCAN/IL/2000/LRC-L792 – lanes Li1 and MHOM/TN/1980/IPT1 - Li2) gave a shorter 361 bp product.

### Analysis of k26 repeat region of the HASPB gene in *L. donovani*

**Figure 3:**
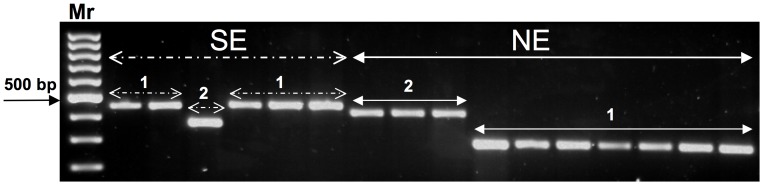
Analysis of Ethiopian *Leishmania donovani* strains by k26 - PCR and agarose gel electrophoresis. PCR products were separated by electrophoresis in 2% agarose gels and stained with ethidium bromide. Southern Ethiopian (SE): 450 bp amplicon −1 and 360 bp amplicon −2. Northern Ethiopian (NE): 290 bp amplicon −1 and 410 bp amplicon −2. A 100 bp molecular weight marker (Mr) is shown on either side of the gel. DNAs from *L. donovani* examined by k26 – PCR in order from left to right: DM290, DM317, AM553, DM283, DM291, AM546, DM256, DM257, DM376sp, GR284, DM14, DM297, DM259, DM287, DM299a, DM389.

The k26 - PCR, a *L. donovani* complex specific assay, targets the repeat region of the *HASPB* gene, and was shown to differentiate among *L. donovani* strains based on the size of the PCR product. *L. donovani* strains from East Africa gave products <430 bp, and Indian isolates showed significantly larger products (∼660 bp) [30], [31]. Strains previously examined from Sudan (n = 6) and Ethiopia (n = 2) gave two main products, ∼284 and ∼430 bp, with one Ethiopian isolate in each group. These strains were isolated between 11 to 49 years ago, and mutations in the *HASPB* gene may have occurred over time, or due to repeated passage in culture. In a recent report where five clinical isolates from Ethiopia were examined only one product, ∼290 bp, was observed [31]. Therefore, we decided to examine a large number, n = 63, of recent *L. donovani* strains isolated from VL and HIV – VL co-infected patients in different geographic regions of Ethiopia. Interestingly, four different amplicon sizes were observed: ∼290, ∼360, ∼410 and ∼450 bp (Figure 3). The PCR product sizes for all the strains examined are summarized in Table S1. Surprisingly, there was a good correlation between geographic origin and amplicon size with strains isolated from patients in northwestern Ethiopia giving either ∼290 or ∼410 bp products, and all the strains isolated in southern Ethiopia, except for three, giving ∼450 bp products. Interestingly, the four strains in the k26-410 cluster were isolated from 3 HIV – VL co – infected patients. Two of the strains were obtained from the same patient, one before drug treatment (LDS 373), and one following relapse (DM376). Prior to drug treatment, the parasites cultured from the spleen or bone marrow of the same patient (LDS 373) gave different k26 amplicon sizes, k26-290 or k26-410 respectively, when examined by PCR. The remaining 11 NE strains isolated from HIV – VL patients all grouped in the k26-290 cluster together with all of the strains isolated from HIV negative VL patients.

Endemic regions for VL in northwestern and southern Ethiopia extend into neighboring Sudan and Kenya, respectively. For this reason, it was interesting to see whether AM553 (k26-360), which gave a unique amplicon different from the other southern strains, represented a second group. This strain is from Negele-Borena close to the border with northwest Somalia and northeast Kenya. Seven *L. donovani* strains from Kenya were screened by k26 – PCR. All of the Kenyan strains produced amplicons larger than the Ethiopian *L. donovani* strains examined here. Of these, 6/7 Kenyan strains gave products ∼500 bp and 1/7 strains gave a product of ∼650 bp. Both Sudanese reference strains examined in this study gave a 290 bp PCR fragment, similar to that previously reported [30], and belong to the k26-290 cluster (data not shown).

**Figure 4:**
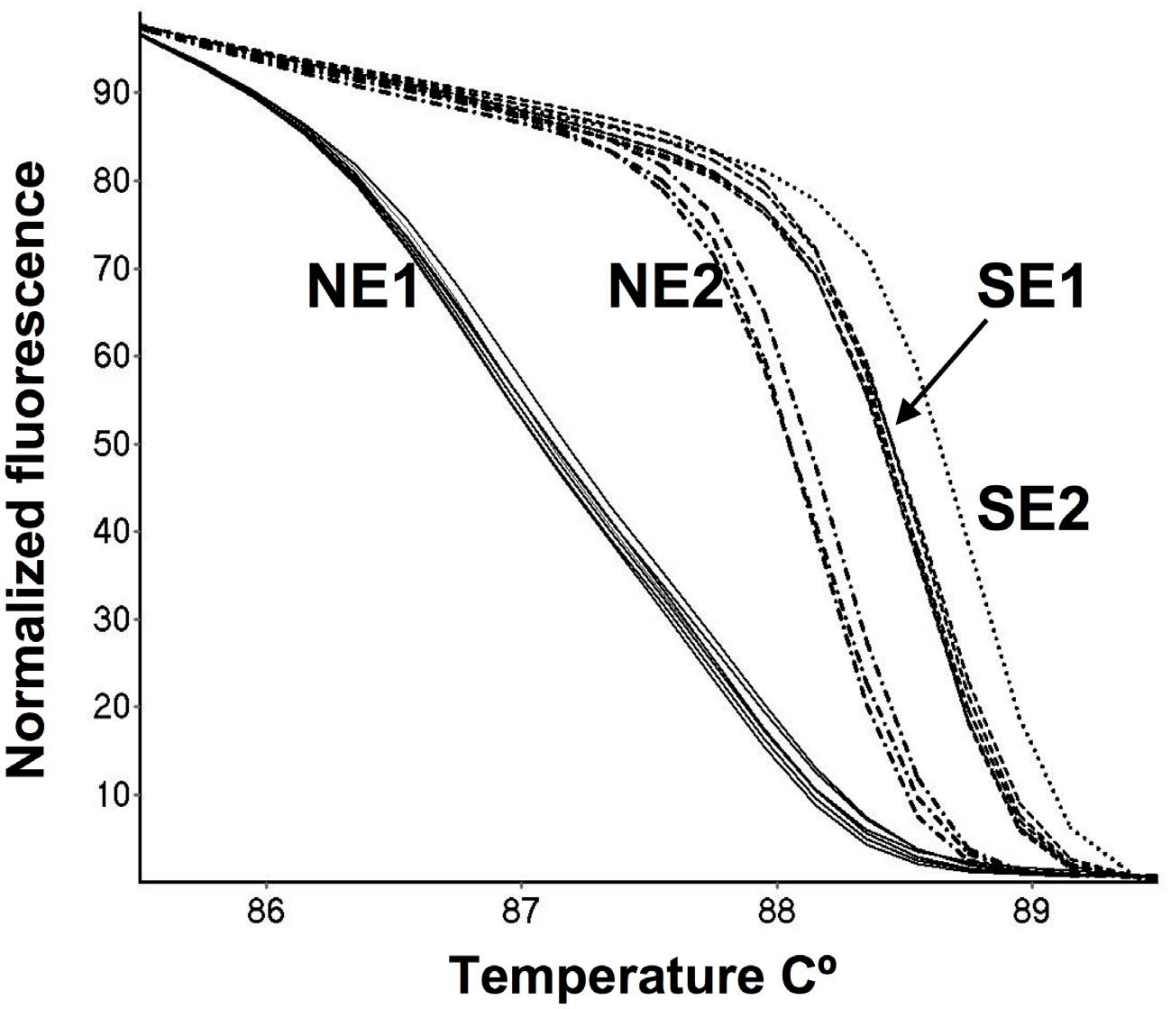
High resolution melting (HRM) curves for k26 - PCR amplicons of Ethiopian *Leishmania donovani*. Plot of normalized fluorescence versus temperature of strains from southern Ethiopia: SE1, 450 bp and SE2, 360 bp. Northern Ethiopia, NE 1, 290 bp and NE2, 410 bp. Strains shown in figure: SE1 – DM283, DM290, DM291, DM317, AM546 ; SE2 - AM553; NE1 - DM259, GR284, DM287, DM 297, DM 299a and DM14 and NE2 - DM256, DM257, DM376spl.

HRM analysis is a rapid and inexpensive method for detecting polymorphisms in double stranded DNA that can potentially distinguish between single base differences. This technique was used in conjunction with k26 - PCR to examine the Ethiopian strains. Typical results are shown in Figure 4. These results show that this technique can be used to rapidly and easily distinguish between the groups found in Ethiopia (k26-290, -360, -410 and -450).

**Figure 5:**
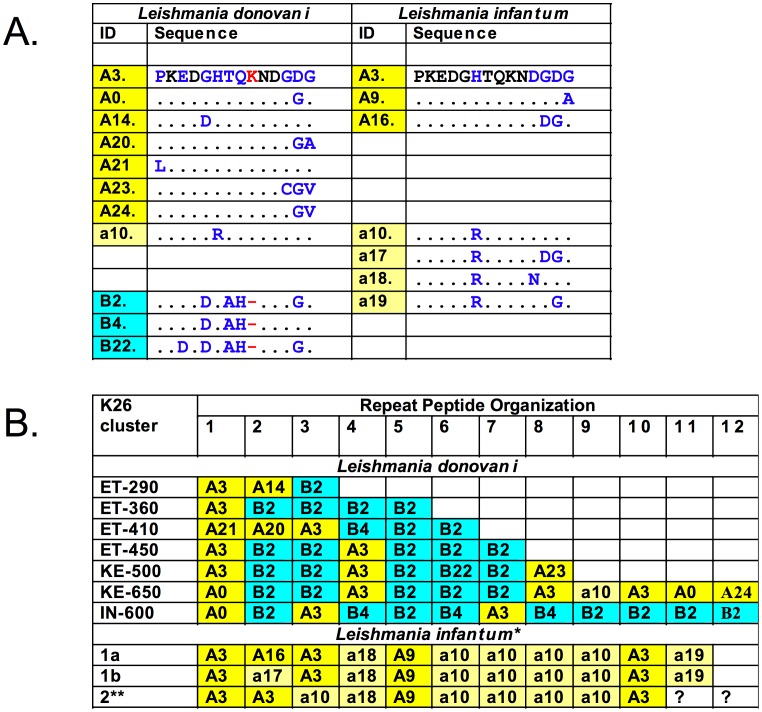
Amino acid sequences and organization of the *Leishmania donovani* complex HASPB repeat region. Panel A. Comparison of peptide repeats motif sequences found in *L. infantum* and *L. donovani*. Panel B. Bar code of peptide motif organization for the k26 repeat region of HASPB in *Leishmania donovani* complex from different geographic regions of the Old World. Peptides A (bright yellow) −14 amino acid peptides found both in *L. donovani* and/or *L. infantum*; Peptides a (banana yellow) −14 amino acid peptides found primarily in *L. infantum* containing the arginine (R) substitution at position 6; Peptides B (blue) −13 amino acid peptides found in *L. donovani*. Peptide A3 found in both species was chosen as the reference sequence to which the amino acid sequences of all the other peptides are compared: Single amino acid abbreviations in blue indicates a substitution, in red a deletion, and in black conserved; (−) missing amino acid, (.) conserved amino acid. Peptides numbers from 0–19 have amino acid sequences identical to those reported by Maroof et al. [29]; those with numbers ≥ 20 are new sequences described in this study.

The k26 - PCR and HRM results suggested that there is little size and DNA sequence variation within each Ethiopian *L. donovani* geographic cluster. This was confirmed by DNA sequencing of 15 amplicons (Genbank accession Nos.: JX088380 - JX088392, JX294866, JX294867) from samples belonging to the four Ethiopian clusters. Analysis of the amino acid sequences (Figure 5) showed that the HASPB repeat region for each *L. donovani* group in Ethiopia is comprised of two motifs, A and B, 14 and 13 amino acids long respectively. These motifs are further distinguished by the amino acids *GHTQK* and *DHAH* present in the central region of each peptide (shown in italics). Two peptides, A3 (PKED*GHTQK*NDGDG) and B2 (PKED*DHAH*NDGGG), comprise 81% of the peptides found in the repeat region, and represent 62.5 and 92.3%, respectively, of the A (Figure 5, yellow) and B (Figure 5, blue) motifs observed in the Ethiopian strains. Several amino acid substitutions, primarily at positions 5, 12–14 of peptide A3 or positions 3 & 12 of peptide B2, also occur in each of the motifs (Figure 5A). As expected, the number of repeats correlates with the size of the PCR amplicon (Figure 5B), however the organization of the peptide repeats is different for each cluster, and doesn't appear to be due to simple DNA duplication or deletion. The order of the peptide motifs observed for each of the Ethiopian cluster can be thought of as a bar code specific for that region.

Kenyan and Indian *L. donovani* strains produce larger k26 -PCR amplicons than the Ethiopian strains (this study and [30], [31]). As such it was interesting to sequence these products and determined the peptide composition and organization of the HASPB repeat region (Genbank accession No.: JX294868–JX294870). This region in the Kenyan and Indian *L. donovani* strains is also comprised of the same peptide motifs, A and B, found in the Ethiopian strains. Several amino acid substitutions (A0, a10, A23, A24 and B22), not observed in HASPB of the Ethiopian strains, are found in these parasites (Figure 5), but A3 and B2 still comprise a majority of the sequences observed. Together, these two peptides comprise 75 and 66.6% of the sequences found in the Kenyan and Indian strains, respectively. The combined percentage of peptides A3 and B2 for the Indian *L. donovani* strain described here is similar to that reported for other Indian isolates, 59.7% [29], even though additional peptide sequences, not observed in our study, were found in the latter isolates (Table S2). However, if the motif A (yellow) or B (blue), rather than the specific peptide sequence, is examined, then a similarity in organization of the repeats, ABBABBB, in the Kenyan and Ethiopian-450 k26 clusters is readily apparent.

The repeat region of the *L. chagasi* (syn = *L. infantum*) HASPB gene was previously characterized and cloned; and has been used in serological assays for VL with mixed results [19], [21], [22], [23], [24], [25], [26]. Sequences for *L. infantum* strains from Brazil, France, Greece, Iran, and Spain (Genbank accession Nos.: AF131228.1, EF504256.1, EF504255.1, EF504258.1, EF504257.1, DQ192034.1, and FR796455.1) show that the HASPB repeat region is only comprised of 14 amino acid peptide repeats. Two peptides A3 (PKED*GHTQK*NDGDG) and a10 (PKED*GRTQK*NDGDG) comprise a majority of the *L. infantum* k26 repeats. Peptide A3 is identical to the peptide found in the *L. donovani* repeat region, while peptide a10 only differs from peptide A3 by substitution of arginine for histidine at position 6 (underlined), and should be considered a member of the peptide A archetype family. However, the latter peptide, a10, does not appear to be very common in East African *L. donovani*, appearing only once among all the parasites examined to date. Conversely, the *L. donovani* 13 amino acid peptide B archetype family, exemplified by PKED*DHAH*NDGGG (peptide B2) and other B peptides (Figure 5 and Table S2), was not present in any of the seven *L. infantum* sequences examined above, as well as six additional strains from Israel (data not shown). However, peptide B8 (Table S2) belonging to the B family archetype appears once in a *L. infantum* strain previously analyzed [29]. HASPB repeat region in fifteen *L. infantum/L. chagasi* strains contained almost exclusively peptides belonging to the A family archetype. The organization of peptide motifs was very similar for all the *L. infantum* strains where sequence data was available (Figure 5). However, most of the isolates analyzed belong to clusters 1a and 1b [30] which both give 626 bp amplicons by k26 - PCR.

**Figure 6:**
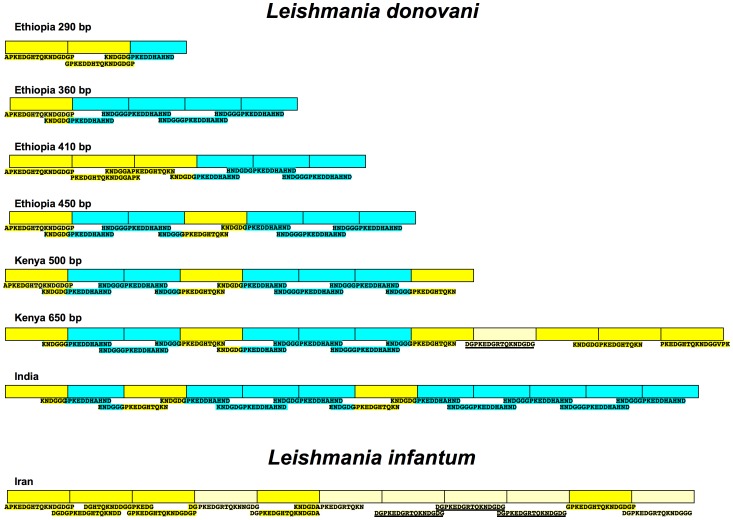
Predicted B-cell epitopes in HASPB repeat regions of *Leishmania donovani* and *L. infantum*. Bar codes, see Figure 5B for legend, showing the A(a) or B motif organization of the repeat region for East African and Indian *L. donovani,* and for *L. infantum* cluster 1a. Non-overlapping B-cell epitopes, 16 amino acids long with a threshold ≥ 0.8, were predicted using a recurrent artificial neural network (ABCpred server [36]). The position of each epitope is indicated under the respective k26 – PCR product cluster bar code. The B-cell epitope recognized by infected canine visceral leishmaniasis sera, DGPKEDGRTQKNDGDG , is underlined.

The HASPB repeat region of *L. donovani* and *L. infantum* strains is predicted to contain multiple linear B-cell epitopes using two different programs (Figure 6, and data not shown [35], [36]). Most of the predicted epitopes (16 amino acids long, threshold ≥ 0.8 out of 1.0) in the *L. donovani* k26 clusters (East Africa and India) span motif junctions (A|A, A|B, B|A or B|B, 84%) with a unique *L. donovani* sequence, K/HNDGD/GG | PKEDDHAHND, accounting for 32/50 (64%) of these epitopes (Figure 6). This sequence is even more predominant, 80–100% of the predicted epitopes, in the southern Ethiopian, Kenyan and Indian *L. donovani* k26 clusters which contain multiple B motifs. This epitope is not seen in the *L. infantum* k26 repeat region, as the B motif is rarely observed in this species. Instead most of the predicted B-cell epitopes, 75%, contain the complete 14 amino acid A motifs, with only a few centered at the A | A motif junctions. Several of the predicted *L. infantum* B-cell epitopes are also found in *L. donovani*.

In this study we examined 63 recent strains isolated from Ethiopian VL patients in different regions of the country. All the parasites were shown to be *L. donovani* by three techniques, confirming previous findings that this species, not *L. infantum*, is responsible for VL in Ethiopia. Interestingly, we found that parasites from northwestern and southern Ethiopia could be easily distinguished based on the size of the k26 – PCR amplicons or their corresponding HRM curves. A similar clustering into two major populations by geographic origin was first reported using multiple microsatellite markers that grouped Sudanese and northwestern (Metema, Humera and Belessa) strains separately from Kenyan and southern strains (Negele-Borena and Konso) [15]. Clustering into genetically separate populations is perhaps, expected, since the primary sand fly vectors, *P. orientalis* and *P. martini*, and habitats are different for the two regions. Other differences between parasites isolated from patients in these two regions, such as sensitivity to paromomycin, have been reported [38]. Interestingly, parasites from northwestern Ethiopia could be divided into additional groups based on the k26 amplicon size, 290 bp and 410 bp. All the Sudanese parasites examined so far gave PCR products similar in size to parasites from northwestern Ethiopia (this study, [13], [30], and data not shown). While the k26-290 group contained isolates from both VL (n = 25) and HIV-VL co-infected (n = 11) patients, the k26-410 group only contained strains from HIV-VL co-infected patients (n = 3). Of the latter isolates, 3/4, were previously analyzed using microsatellite markers [15], and belong to subpopulation B2. Interestingly, this subpopulation was postulated to represent one parent strain of putative hybrid/mixed genotypes.

Different k26 – PCR products were also found when parasite strains from southern Ethiopia were analyzed, k26 −290, −360 and −450. All of the strains examined except three (AM422, AM452, and AM553) produced a 450 bp amplicon. Since microsatellite analysis grouped southern Ethiopian and some Kenyan parasites together [15], and *P. martini* is the primary vector involved in the *L. donovani* transmission in these regions [9], we decided to examine several Kenyan strains by k26 – PCR. Surprisingly, the k26 amplicons for all the Kenyan parasites tested were larger (∼500 and ∼650 bp) than those found for the south Ethiopian isolates, and similar in size to Indian *L. donovani* parasites (this study and [30]). Thus, there doesn't seem to be a direct correlation between the size of the k26 amplicon, and the microsatellite cluster to which the strain belongs. It is not clear whether the two southern Ethiopian strains that gave the 290 bp PCR product represent a third group present in this region, are a result of human migration or are due to culture contamination. The k26 DNA sequence for these strains is identical to the other 290 bp Sudanese and northern Ethiopian strains examined (Table S1, and data not shown). Interestingly, one of the strains, AM422, originates from the Omo Valley where transmission by both vectors may occur, and is close to Sudan. More work is needed to determine whether there is a direct correlation between the parasite vector and k26 genotype, as HASPB plays a role in parasite differentiation and localization in the sand fly [20]. At this time it is not clear why *L. donovani* strains from different regions in East Africa show variations in the k26 – PCR fragment size, or the factors responsible for the size polymorphism, however this technique appears to be useful for rapid mapping of strain origin on a large scale.

The HASPB1 protein is a potential vaccine candidate, as well as a diagnostic antigen [19], [21], [22], [23], [24], [25], [26], [27], [28], [29], [39], [40]. However, serodiagnostic assays using the HASPB1 protein or k26 repeat region as antigen have produced conflicting results. While assays using sera from canine or human VL caused by *L. infantum* give consistently high sensitivity (94–100%) and specificity (100%) [23], [24], [39], similar assays using VL sera from patients in India and Sudan showed variable sensitivity (India −21.3 and 38%; Sudan −92 and 93.5%) [21], [22], [25], [26]. Assay specificity in latter studies was consistently high (80–100%). Interestingly, the assays showing low sensitivity in Indian VL patients used the *L. infantum* k26 antigen [21], [22], while assays demonstrating high sensitivity in Sudanese VL patients used the *L. donovani* antigen [25], [26]. The B-cell epitopes recognized by serum antibodies in the HASPB1 repeat region have not been extensively analyzed, though one study reported that the 17 amino acid peptide, *G*DGPKEDGRTQKNDGDG from *L. infantum* reacted strongest with canine VL sera [41]. Interestingly, when putative linear B-cell epitopes in the *L. infantum* k26 repeat region were predicted (Figure 6) using a recurrent artificial neural network (ABCpred server [36]) a peptide, DGPKEDGRTQKNDGDG, 16 amino acids in length, and identical in 16/17 amino acid residues to the peptide recognized by canine sera above, ranked first with a score of 0.88 out of 1.0. This peptide includes the 14 amino acid motif (a10 – PKEDGRTQKNDGDG) frequently found in *L. infantum* (Figures 5 and 6), but rarely in *L. donovani* strains (this study and [29]). The a10 motif was predicted to be a B-cell epitope (score = 0.81). On the other hand, none of the peptide motifs (B2, B4 and B22; PKE/DDDHAHNDGG/DG) unique to *L. donovani* rk26 are found in *L. infantum*, and combinations of these motifs generated *L. donovani* B-cell epitopes giving the highest scores (e.g., KNDGDGPKEDDHAHND, 0.88; HNDGGGPKEDDHAHND, 0.87; HNDGDGPKEDDHAHND, 0.87; and data not shown). It will be interesting to see if better sensitivity and specificity can be obtained using either single antigen or mixtures of recombinant k26 antigens produced from the *L. donovani* strains responsible for local disease in Ethiopia and Sudan. This work is in progress.

HASPB1 is differentially expressed by metacyclic promastigotes and intracellular amastigotes [42]. Immunization of BALB/c mice with *L. donovani* HASPB1, even in the absence of adjuvant, generates a protective CD8+ T-cell response via an immune complex-mediated complement activation involving natural antibodies against a challenge with this parasite [27], [28]. The CD8+ T-cell epitopes were shown to reside in both the conserved and repeat regions of the protein [29]. While a role for HASPB in the development of metacyclic promastigotes was demonstrated [20], the function of these proteins in amastigotes is not yet clear. Interestingly, an orthologous protein, O-HASP, from *L. (Viannia) braziliensis* showed considerable genetic polymorphism in the repeat region among clones isolated from individual patients [43], and it was postulated that genetic variation may play a role in immune recognition. A similar phenomenon appears to occur in Old World *Leishmania* causing VL, as one report suggests that clonal variation is present in HASPB of Indian *L. donovani* strains [29]. However, DNA sequencing of 21 clones from four Ethiopian strains (k26-290 bp) did not identify any polymorphism in the repeat region of this protein (data not shown).

In summary, we show that the number, order and arrangement of the *L. donovani* k26 repeat region of the HASPB protein varies among strains from different geographic regions, and that the repeat motifs are different from those observed for *L. infantum*. The role that this genetic variation plays in the interaction with the host and vector is not clear and should be investigated further.

## Supporting Information

Table S1 ***L. donovani*** **strains used in this study.**
(DOCX)

Table S2 **Summary of peptides found in the HASPB repeat region of parasites belonging to the** ***Leishmania donovani*** **complex.**
(DOC)
