# Supplementary material for: Polymorphism in the HASPB Repeat Region of East African Leishmania donovani Strains
Source: PLoS Negl Trop Dis. 2013 Jan 24;7(1):e2031. doi: 10.1371/journal.pntd.0002031 (PMC3554577; doi:10.1371/journal.pntd.0002031)
Supplement: Table S1 — L. donovani strains used in this study. (DOCX) [file pntd.0002031.s001.docx]

Table S1. *L. donovani* strains used in this study.

| WHO code | Pathologya | k26 - PCR approximate product size (bp) | Microsatellite Clusterb | Likely place/locality of infection | GenBank accession No. | Sourcec |
| --- | --- | --- | --- | --- | --- | --- |
| MHOM/ET/2009/AM421 | HIV/VL-Sp | 450 | nd | Konso, SE | nd | This study |
| MHOM/ET/2009/AM422 | HIVVL/Sp | 290 | nd | Omo Valley, SE | JX294866 | This study |
| MHOM/ET/2009/AM440 | VL/Sp | 450 | nd | Negele-Borena, SE | nd | This study |
| MHOM/ET/2009/AM452 | VL/Sp | 290 | nd | Konso, SE | JX294867 | This study |
| MHOM/ET/2009/AM459 | VL/Sp | 450 | nd | Konso, SE | nd | This study |
| MHOM/ET/2009/AM486 | VL/Sp | 450 | nd | Konso, SE | nd | This study |
| MHOM/ET/2010/AM546 | VL/Sp | 450 | nd | Konso, SE | JX088391 | This study |
| MHOM/ET/2010/AM548 | VL/Sp | 450 | nd | Konso, SE | nd | This study |
| MHOM/ET/2010/AM551 | VL/Sp | 450 | nd | Konso, SE | nd | This study |
| MHOM/ET/2010/AM552 | VL/Sp | 450 | nd | Negele-Borena, SE | nd | This study |
| MHOM/ET/2010/AM553 | VL/Sp | 360 | nd | Negele-Borena, SE | JX088392 | This study |
| MHOM/ET/2010/AM554 | VL/Sp | 450 | nd | Negele-Borena, SE | nd | This study |
| MHOM/ET/2010/AM560 | VL/Sp | 450 | nd | Konso, SE | nd | This study |
| MHOM/ET/2010/AM563 | VL/Sp | 450 | nd | Negele-Borena, SE | nd | This study |
| MHOM/ET/2008/DM283 | VL/Sp | 450 | SE/KE; NB/KE; NB | Konso, SE | JX088389 | 1 |
| MHOM/ET/2008/DM284 | VL/Sp | 450 | SE/KE; NB/KE; NB | Negele-Borena, SE | nd | 1 |
| MHOM/ET/2008/DM285 | VL/Sp | 450 | SE/KE; KO; KO+KE+NB | Konso, SE | nd | 1 |
| MHOM/ET/2008/DM288 | VL/Sp | 450 | SE/KE; KO; KO+KE+NB | Konso, SE | nd | 1 |
| MHOM/ET/2008/DM289 | VL/Sp | 450 | SE/KE; KO; KO | Konso, SE | nd | 1 |
| MHOM/ET/2008/DM290 | VL/Sp | 450 | SE/KE; KO; KO | Konso, SE | JX088387 | 1 |
| MHOM/ET/2008/DM291 | VL/Sp | 450 | SE/KE; NB/KE; NB | Negele-Borena, SE | JX088390 | 1 |
| MHOM/ET/2008/DM292 | VL/Sp | 450 | SE/KE; NB/KE; NB | Negele-Borena, SE | nd | 1 |
| MHOM/ET/2009/DM317 | VL/Sp | 450 | SE/KE; KO; KO | Konso, SE | JX088388 | 1 |
| MHOM/ET/2009/GR284 | VL/BM | 290 | nd | N/Gondar – Metema, NE | nd | This study |
| MHOM/ET/2009/GR300 | VL/BM | 290 | nd | Humera, NE | nd | This study |
| MHOM/ET/2009/GR353 | VL/Sp | 290 | nd | N/Gondar - Metema, NE | nd | This study |
| MHOM/ET/2009/GR356 | VL/Sp | 290 | nd | N/Gondar - Metema, NE | nd | This study |
| MHOM/ET/2009/GR358 | VL/BM | 290 | nd | Humera, NE | nd | This study |
| MHOM/ET/2009/GR361 | VL/BM | 290 | nd | N/Gondar - Metema, NE | nd | This study |
| MHOM/ET/2010/GR363sp | HIV/VL - Sp | 290 | nd | Humera, NE | nd | This study |
| MHOM/ET/2010/GR363sk | HIV/VL - Sk | 290 | nd | Humera, NE | nd | This study |
| MHOM/ET/2010/GR364sp | HIV/VL -Sp | 290 | nd | Humera, NE | nd | This study |
| MHOM/ET/2010/GR364sk | HIV/VL - Sk | 290 | nd | Humera, NE | nd | This study |
| MHOM/ET/2010/GR378 | VL/BM | 290 | nd | Humera, NE | JX088381 | This study |
| MHOM/ET/2010/GR379 | VL/Sp | 290 | nd | Metema, NE | nd | This study |
| MHOM/ET/2010/GR383 | VL/Sp | 290 | nd | Humera, NE | nd | This study |
| MHOM/ET/2010/GR412 | VL/Sp | 290 | nd | Humera, NE | nd | This study |
| MHOM/ET/2010/GR428 | VL/BM | 290 | nd | Humera, NE | nd | This study |
| MHOM/ET/2010/GR429 | VL/BM | 290 | nd | Humera, NE | nd | This study |
| MHOM/ET/2010/GR430 | VL/Sp | 290 | nd | Humera, NE | nd | This study |
| MHOM/ET/2010/GR433 | VL/Sp | 290 | nd | Humera, NE | nd | This study |
| MHOM/ET/2010/GR435 | VL/BM | 290 | nd | Humera, NE | nd | This study |
| MHOM/ET/2010/GR440 | VL/BM | 290 | nd | Humera, NE | nd | This study |
| MHOM/ET/2010/GR459 | VL/BM | 290 | nd | Humera, NE | nd | This study |
| MHOM/ET/2009/LDS 373bm | HIV/VL - BM | 410 | nd | N/Gondar/Humera, NE | nd | This study |
| MHOM/ET/2009/LDS 373sp | HIV/VL-Sp | 290 | nd | N/Gondar/Humera, NE | nd | This study |
| MHOM/ET/2009/LDS 005/09 | HIV/VL-Sp | 290 | nd | Humera, NE | nd | This study |
| MHOM/ET/2010/LDS 148/10 | HIV/VL-Sp | 290 | nd | Humera, NE | nd | This study |
| MHOM/ET/2007/DM14 | VL/Sp | 290 | NE/SD; A; A1 | Gondar, NE | JX088385 | 1 |
| MHOM/ET/2008/DM256 | HIV/VL-Sp | 410 | NE/SD; B; B2 | N/Gondar/Humera, NE | JX088385 | 1 |
| MHOM/ET/2008/DM257 | HIV/VL-Sp | 410 | NE/SD; B; B2 | Humera, NE | JX088386 | 1 |
| MHOM/ET/2008/DM259 | VL/Sp | 290 | NE/SD; A; A3 | Humera, NE | JX088384 | 1 |
| MHOM/ET/2008/DM276 | VL/BM | 290 | NE/SD; A; A1 | Humera, NE | nd | 1 |
| MHOM/ET/2008/DM278 | VL/Sp | 290 | NE/SD; A; A3 | Gondar, NE | nd | 1 |
| MHOM/ET/2008/DM286 | VL/Sp | 290 | NE/SD; A; A1 | N/Gondar, NE | nd | 1 |
| MHOM/ET/2008/DM287 | VL/Sp | 290 | NE/SD; B; B1 | East Sudan/Humera, NE | nd | 1 |
| MHOM/ET/2008/DM294 | VL/Sp | 290 | NE/SD; A; A3 | Metema, NE | nd | 1 |
| MHOM/ET/2008/DM297 | VL/BM | 290 | NE/SD; A; A2 | Debub Wollo/Humera, NE | JX088383 | 1 |
| MHOM/ET/2008/DM299a | HIV/VL-Sp | 290 | NE/SD; B; B1 | N/Gondar - Abdurafi, NE | JX088380 | 1 |
| MHOM/ET/2009/DM376sp | HIV/VL-Sp | 410 | NE/SD; B; B2 | N/Gondar/Humera, NE | nd | 1 |
| MHOM/ET/2008/DM389 | HIV/VL-Sp | 290 | NE/SD; B; B1 | Gondar, NE | nd | 1 |
| MHOM/ET/2009/DM446 | HIV/VL-Sp | 290 | NE/SD; A; A3 | Gondar, NE | nd | 1 |
| MHOM/ET/2009/DM451 | HIV/VL-Sp | 290 | NE/SD; A; A1 | Gondar, NE | nd | 1 |
| MHOM/SD/1968/1S cl2 | VL | 290 | NE/SD; A; A2 | ni | nd | 2 |
| MHOM/SD/??/Khartoum | VL | 290 | nd | ni | nd | 2 |
| MHOM/KE/83/NLB 189 | PKDL | 500 | SE/KE; NB/KE; KE India 2 | ni | nd | 1 |
| MHOM/KE/54/LRC-L53 | VL | 500 | SE/KE; NB/KE; KE India 2 | ni | nd | 1 |
| MHOM/KE/55/LRC-L53 | VL | 500 | SE/KE; NB/KE; KE India 2 | ni | JX294868 | 1 |
| MHOM/KE/73/MRC74 | VL | 500 | SE/KE; NB/KE; KO+NB+KE  India 2 | ni | nd | 1 |
| IMAR/KE/62/LRC-L57 | VL | 500 | SE/KE; NB/KE; KE India 2 | ni | nd | 1 |
| MHOM/KE/??/LRC-L445 | VL | 500 | SE/KE; NB/KE; KE India 2 | ni | JX294869 | 1 |
| MHOM/KE/85/NLB 323 | VL | 650 | SE/KE; NB/KE; KE India 2 | ni | nd | 1 |
| MHOM/IN/1980/DD8 | VL | 600 | India 1 | ni | nd | 2 |
| MHOM/IN/2002/BHU32 | VL | 600 | India 1 | ni | JX294870 | 2 |

Footnotes: aVL - visceral leishmaniasis; HIV/VL – HIV plus visceral leishmaniasis co-infection; PKDL: Post Kala-azar Dermal Leishmaniasis; Sp – spleen isolate; Sk – skin isolate and BM – bone marrow. nd – not determined; ni - no information; NE – north Ethiopia; SE – south Ethiopia. b 1. Gelanew T, Kuhls K, Hurissa Z, Weldegebreal T, Hailu W, et al. (2011) Inference of population structure of *Leishmania donovani* strains isolated from different Ethiopian visceral leishmaniasis endemic areas. PLoS Negl Trop Dis 4: e889; 2. Alam MZ, Kuhls K, Schweynoch C, Sundar S, Rijal S, et al. (2009) Multilocus microsatellite typing (MLMT) reveals genetic homogeneity of *Leishmania donovani* strains in the Indian subcontinent. Infection, Genetics and Evolution 9: 24-31. c 1. Gelanew T, et al. (2011). PLoS Negl Trop Dis 4: e889; 2. LRC – Lionel Schnur, *Leishmania* Reference Centre, Hebrew University of Jerusalem, Israel.
