# Supplementary material for: Polymorphism in the HASPB Repeat Region of East African Leishmania donovani Strains
Source: PLoS Negl Trop Dis. 2013 Jan 24;7(1):e2031. doi: 10.1371/journal.pntd.0002031 (PMC3554577; doi:10.1371/journal.pntd.0002031)
Supplement: Table S2 — Summary of peptides found in the HASPB repeat region of parasites belonging to the Leishmania donovani complex. (DOC) [file pntd.0002031.s002.doc]

Table S2. HASPB peptide repeats *Leishmania donovani* complex

| ***Leishmania donovani*** | | | ***Leishmania infantum*** | | |
| --- | --- | --- | --- | --- | --- |
| ID | Sequence | Source* | ID | Sequence | Source* |
| A3 | **PKEDGHTQKNDGDG** |  | A3 | **PKEDGHTQKNDGDG** |  |
| A0 | ............G. | 1, 2 |  |  |  |
| A1 | ........N...D. | 2 |  |  |  |
| A3 | **.............** | 1, 2 | A3 | **..............** | 1, 2 |
| A7 | .E..........G. | 2 |  |  |  |
| A9 | .............A | 2 | A9 | .............A | 2, 3 |
| A13 | ..K........... | 2 |  |  |  |
| A14 | ....D......... | 1, 2 |  |  |  |
|  |  |  | A16 | ...........DG. | 1, 2 |
| A20. | ............GA | 1 |  |  |  |
| A21 | L............. | 1 |  |  |  |
| A23. | ...........CGV | 1 |  |  |  |
| A24. | ............GV | 1 |  |  |  |
|  |  |  |  |  |  |
| a10 | .....R........ | 1, 2 | a10 | .....R........ | 1, 2, 3 |
|  |  |  | a17 | .....R.....DG. | 2, 3 |
|  |  |  | a18 | .....R....N... | 2, 3 |
|  |  |  | a19 | .....R......G. | 2, 3 |
|  |  |  |  |  |  |
| B2 | ....D.AH-...G. | 1, 2 |  |  |  |
| B4 | ....D.AH-..... | 1, 2 |  |  |  |
| B5 | ....D.AH-...N. | 2 |  |  |  |
| B6 | ....D.AH-...GC | 2 |  |  |  |
| B8 | ....D.AH-S..G. | 2 | B8 | ....D.AH-S..G. | 2 |
| B15 | ..K.D.AH-..... | 1 |  |  |  |
| B22 | ..D.D.AH-...G. | 1 |  |  |  |

* 1. This study; 2. Maroof A, Brown N, Smith B, Hodgkinson MR, Maxwell A, et al. (2012) Therapeutic vaccination with recombinant adenovirus reduces splenic parasite burden in experimental visceral leishmaniasis. J Infect Dis 205: 853-863; 3. GenBank accession numbers for *L. infantum* k26 sequences: AF131228.1, EF504256.1, EF504255.1, EF504258.1, EF504257.1, DQ192034.1, and FR796455.1
